# Dr. R. W. Read

**Published:** 1909-09

**Authors:** 


Dr. R. W. Read, of Texarkana, died August 2 (ult.), aged 73. He was one of the ablest, best known and most popular physicians in Texas, and his death will be deplored by a very wide circle of attached friends. He was exceptionally pure in his professional, as in his social life, being of the old school of ethics. He was a native of Alabama, but became a Texan at the age of 18. Matriculated at Tulane in 1856; graduated M. D. from Medical Department University of Pennsylvania in 1858. Entered the Confederate States Army in 1862, being appointed surgeon (major). In this capacity he served in the field until the war ended. In 1859 he married Miss Elisabeth Kimball. Of the nine children born, six survive him, Mrs. S. A. Collom (wife of Dr. Collom of Texarkana, Texas), one is the wife of Dr. L. H. Bush, of Huntsville, one the wife of Judge R. L. Penn, of Austin, and the other is Mrs. Whippert, of Bryan. His son—Dr. W. K. Read—his associate in practice, at Texarkana, succeeds him, and Mr. A. C. Reed is a druggist at Little Rock, Ark. Dr. Read's death even at so advanced an age is a distinct loss to the profession and to the State, for he was a man of deeds, not words.

